# To hydrate or not to hydrate? The effect of hydration on survival, symptoms and quality of dying among terminally ill cancer patients

**DOI:** 10.1186/s12904-021-00710-9

**Published:** 2021-01-12

**Authors:** Chien-Yi Wu, Ping-Jen Chen, Tzu-Lin Ho, Wen-Yuan Lin, Shao-Yi Cheng

**Affiliations:** 1 Department of Family Medicine, Kaohsiung Medical University Hospital, Kaohsiung Medical University, Kaohsiung, Taiwan; 2 Division of Geriatrics and Gerontology, Kaohsiung Medical University Hospital, Kaohsiung Medical University, Kaohsiung, Taiwan; 3 Marie Curie Palliative Care Research Department, Division of Psychiatry, University College London, London, UK; 4 Department of Family Medicine, College of Medicine and University Hospital, National Taiwan University, 7 Chung-Shan South Road, Taipei, 100 Taiwan; 5 Department of Family Medicine, China Medical University Hospital, Taichung, Taiwan; 6 School of Medicine, College of Medicine, China Medical University, Taichung, Taiwan

**Keywords:** Artificial hydration, Cancer, Survival, Quality of dying, Palliative care

## Abstract

**Background:**

Artificial nutrition and hydration do not prolong survival or improve clinical symptoms of terminally ill cancer patients. Nonetheless, little is known about the effect of artificial hydration (AH) alone on patients’ survival, symptoms or quality of dying. This study explored the relationship between AH and survival, symptoms and quality of dying among terminally ill cancer patients.

**Methods:**

A pilot prospective, observational study was conducted in the palliative care units of three tertiary hospitals in Taiwan between October 2016 and December 2017. A total of 100 patients were included and classified into the hydration and non-hydration group using 400 mL of fluid per day as the cut-off point. The quality of dying was measured by the Good Death Scale (GDS). Multivariate analyses using Cox’s proportional hazards model were used to assess the survival status of patients, the Wilcoxon rank-sum test for within-group analyses and the Mann-Whitney U test for between-groups analyses to evaluate changes in symptoms between day 0 and 7 in both groups. Logistic regression analysis was used to assess the predictors of a good death.

**Results:**

There were no differences in survival (*p* = 0.337) or symptom improvement between the hydration and non-hydration group, however, patients with AH had higher GDS scores.

**Conclusions:**

AH did not prolong survival nor significantly improve dehydration symptoms of terminally ill cancer patients but it may influence the quality of dying. Communication with patients and their families on the effect of AH may help them better prepared for the end-of-life experience.

## Background

Previous studies have found that patients who receive palliative care have a better quality of life (QOL) as well as end-of-life experience [1–3]. In the clinical practice of end-of-life care, terminally ill cancer patients may cease to benefit from oral nutrition and fluids during the very terminal stage [4, 5]. However, many family members and even patients themselves request medical staff to continuously administer artificial hydration (AH) [5–7]. Therefore, medical professionals often encounter an ethical dilemma related to the provision of artificial nutrition and hydration (ANH) [8, 9].

A Taiwanese study found that ANH did not prolong the survival of terminally ill cancer patients [6], and a randomised controlled trial of the influence of AH on terminally ill cancer patients showed no obvious difference in dehydration symptoms, QOL and survival between groups receiving 1 L and 100 ml of fluid daily [10]. In a Japanese study, except for the improvement in membranous dehydration symptoms, hydration provided no benefit, but instead exacerbated fluid overload, induced hypoalbuminemia and failed to correct electrolyte imbalance [11–14]. Therefore, Japanese clinical guidelines do not suggest that medical professionals administer AH routinely if there is no specific need [15]. Indeed, the patient’s condition, fluid overload condition and the attitude of family members are key factors in whether to administer AH [16]. In another Japanese study of over 5000 members of the general population and 800 bereaved family members, 33 to 50% of respondents believed that administering AH to terminally ill patients during the very terminal stage was a part of basic care, with 15 to 31% of respondents believing that AH could relieve symptoms [17]. In a western study, ethnicity played an important role in whether AH was perceived as food or medicine. Ethnic minorities in the United States, such as African Americans, Latinos and Asian Americans (total 66%), were significantly more likely to view AH as food or as both food and medicine than non-Hispanic white subjects (42%) [18]. In an Italian study, patients and their families considered AH as useful medical management, with most preferring the intravenous route, as they thought it could improve clinical conditions and had a positive psychological meaning [7]. Thus, cross-cultural comparison of the role of ANH is both practical and culturally sensitive.

Previous research shows that AH may be more harmful than beneficial to terminally ill cancer patients’ QOL. However, little is known about the influence of AH on patients’ quality of dying, therefore, the primary outcome of this pilot prospective observational study was to investigate the influence of AH on patients’ quality of dying. Also, the relationship between AH and the survival and symptoms were assessed. It was hypothesised that AH would not affect the quality of dyig or improve dehydration symptoms or prolong the survival period.

## Methods

### Study design and participants

A pilot prospective, observational study was conducted in the palliative care units (PCU) of three tertiary hospitals in different cities in Taiwan (National Taiwan University Hospital, Chi-Mei Medical Centre and Kaohsiung Medical University Hospital) between October 2016 and December 2017. These hospitals were selected as they have abundant palliative care experience, as their PCU have been operational for more than 10 years, and they were willing to participate in the clinical observational study. This study was approved by Institutional Review Boards of all three hospitals.

The inclusion criteria for study objects were: (1) patients aged 20 years or older, (2) patients with locally advanced or metastatic cancer (histological, cytological or clinical diagnosis), (3) patients who could not have normal oral intake and (4) patients presenting with malaise and at least one of the following dehydration symptoms, delirium, dry mouth or myoclonus. The exclusion criteria were: (1) patients died less than 24 h after the admission to PCU, (2) patients or their family members declined participation and (3) patients with non-cancer terminal disease. All terminally ill patients in these three PCUs were screened for their eligibility during admission. If the patients met the inclusion criteria, the researchers explained the study purpose and protocol to the patients or their families (proxy) if patients had a conscious disturbance. The patients or their proxy provided written informed consent to participate in the study.

### Outcome measurements

On admission to PCU, the need for AH by intravenous or subcutaneous route was according to clinical evaluation and management. After discussion with patients or their families about AH, the duty physician administered the formulated AH to the patients as required. Patients were classified into the hydration group and the non-hydration group using 400 mL per day as the cut-off point, as the bottle of formulated AH which contains glucose and electrolytes is often 400 mL and is routinely administered to terminally ill cancer patients as a basic fluid supply. The daily hydration volume was calculated together with formulated AH and other fluids for medical purposes, such as antibiotics, albumin or blood transfusion. The two groups were compared to determine the effect of hydration on survival time, symptom relief, Good Death Scale (GDS) and the possible side effects of hydration.

Other recorded variables included patient’s age, gender, primary cancer, Charlson Comorbidity Index, social state, religion, clinical symptoms (including the eating condition by mouth, dyspnoea, fatigue, drowsiness, dry mouth, anorexia, muscle spasm, dysphagia, respiratory tract secretion, oedema, ascites, pleural effusion, bowel obstruction, water intake condition and delirium), blood transfusion, antibiotics use or albumin supply and patient’s functional status as measured by the Eastern Cooperative Oncology Group performance status (ECOG). The eating condition by mouth was classified into reduced but more than a mouthful and less than a mouthful every time while eating. Dyspnoea was classified into no and yes, and the dyspnoea level was further divided into exertional only and at rest. The Integrated Palliative care Outcome Scale (IPOS) was developed to measure the patient’s symptom severity. The ranking was: 0, not at all; 1, slightly; 2, moderately; 3, severely; 4, overwhelmingly; 5, cannot assess. IPOS was used to assess the fatigue, drowsiness, dry mouth and anorexia symptoms. The myoclonus variable evaluated the patient’s worst condition while at rest according to the ranking: 0, none; 1, ≤1 jerk; 2, 2-3 jerks; 3, 4-9 jerks; and 4, ≥10 jerks per 10 s. Dysphagia was divided into no or yes. The respiratory tract secretion variable evaluated the patient’s worst condition, the scale was 0, not audible; 1, only audible at the head of the bed; 2, clearly audible at the foot of the bed, and 3, clearly audible at 6 m from the foot of the bed. Lower extremity oedema was measured by observing the leg with less oedema and ranking 0 as none, 1 as mild (< 5 mm), 2 as moderate (5–10 mm) and 3 as severe (> 10 mm). Ascites and pleural effusion were evaluated by clinical examination or imaging, ranking 0 as none, 1 as physically detectable but asymptomatic and 2 as symptomatic. Bowel obstruction was classified into no or yes. The delirium level was evaluated using item 9 of the Memorial Delirium Assessment Scale (MDAS), decreased or increased psychomotor activity. The clinical symptoms were evaluated by the main healthcare professionals at baseline during admission to PCU and 1 week after enrollment until death.

### Good death scale (GDS)

The GDS was used to evaluate the quality of dying [19–21] according to five domains scored on a 4-point Likert scale: an awareness that one is dying (0, complete ignorance; 3, complete awareness), acceptance of death peacefully (0, complete unacceptance; 3, complete acceptance), honouring of the patient’s wishes (0, no reference to the patient’s wishes; 1, following the family’s wishes alone; 2, following the patient’s wishes alone, and 3, following the wishes of the patient and the family), death timing (0, no preparation; 1, the family alone had prepared; 2, the patient alone had prepared; and 3, both the patient and the family had prepared) and the degree of physical comfort 3 days before death (0, a lot of suffering; 1, suffering; 2, a little suffering; and 3, no suffering). The GDS score, ranging from 0 to 15, was discussed by the experienced palliative care team at the team meeting after each patient died. The score of each item was considered separately and the final score was decided by consensus at the team meeting. The higher the total score, the better the good death status the patient had achieved. The GDS of 68 patients were collected and analysed. A GDS ≧12 indicated a better quality of dying according to the quality indicator set at the National Taiwan University Hospital.

### Statistical analysis

Descriptive analyses were used to assess the differences in demographic characteristics between the two groups. The Kaplan-Meier curve was used to estimate the impact of hydration on survival between the two groups and multivariate analyses using Cox’s proportional hazards model were used to assess the survival time of patients. The Wilcoxon rank-sum test was applied for within-group analyses and the Mann-Whitney U test for between-groups analyses to evaluate changes in symptoms between day 0 and 7 in the hydration and non-hydration group. Finally, logistic regression analysis was used to assess the predictors for patients whose GDS ≧12. The R software was used for the statistical analyses (R Core Team, Foundation for Statistical Computing, Vienna, Austria) and a *p*-value < 0.05 indicated statistical significance.

## Results

**Fig. 1:**
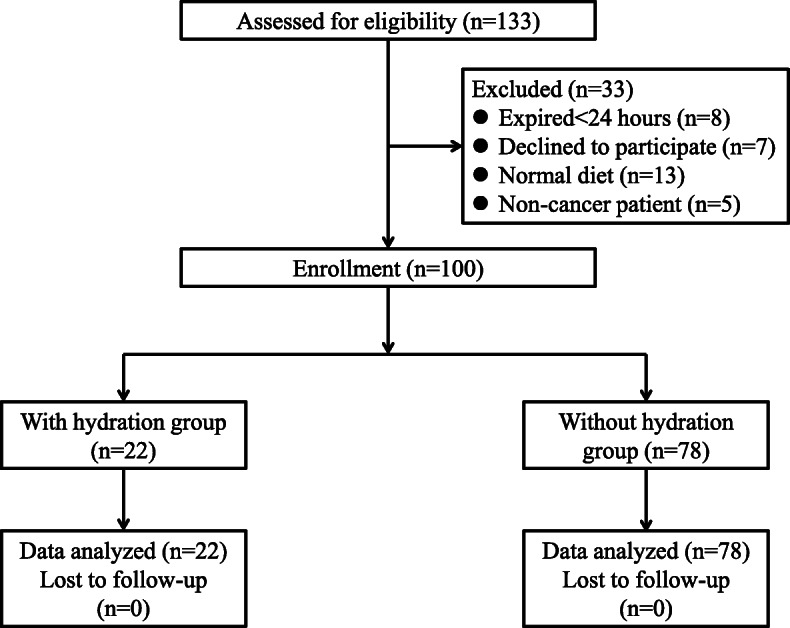
The patient recruitment flow chart

A total of 133 patients were eligible for enrolment in this study, of which, 33 were excluded for the following reasons: 8 patients died within 24 h after admission, 7 patients declined to participate, 13 patients had normal oral intake and 5 patients had a non-cancerous disease. Finally, 100 patients were analysed in this study, 22 in the hydration group and 78 in the non-hydration group. The patient recruitment flow chart is shown in Fig. 1, with the demographic and clinical characteristics of the enrolled patients provided in Table 1.

**Table 1:** Demographic and clinical characteristics of enrolled patients (*n* = 100)

Variable	Total	Hydration		*p*-value
		< 400 ml (*n* = 78)	≧400 ml (*n* = 22)	
**Institution**				0.237
NTUH	43 (43%)	37 (47.4%)	6 (27.3%)	
Chi-Mei	26 (26%)	19 (24.4%)	7 (31.8%)	
KMUH	31 (31%)	22 (28.2%)	9 (40.9%)	
**Age**	69.19 ± 12.89	71.26 ± 11.86	61.86 ± 13.97	0.005
**Gender**				0.630
Female	52 (52%)	42 (53.8%)	10 (45.5%)	
Male	48 (48%)	36 (46.2%)	12 (54.5%)	
**Education**				0.053
≦6 years	49 (49%)	43 (55.1%)	6 (27.3%)	
7 ~ 12 years	35 (35%)	23 (29.5%)	12 (54.5%)	
> 12 years	16 (16%)	12 (15.4%)	4 (18.2%)	
**Marital status**				0.190
Unmarried	6 (6.0%)	5 (6.4%)	1 (4.5%)	
Married	58 (58.0%)	44 (56.4%)	14 (63.6%)	
Widowed	27 (27.0%)	24 (30.8%)	3 (13.6%)	
Separated / divorced	9 (9.0%)	5 (6.4%)	4 (18.2%)	
**Religion**				0.015
Nullifidian	11 (11%)	7 (9.0%)	4 (18.2%)	
Buddhism	24 (24%)	23 (29.5%)	1 (4.5%)	
Christian/ Catholicism	12 (12%)	12 (15.4%)	0 (0%)	
Taoism/Taiwanese folk religion	50 (50%)	34 (43.6%)	16 (72.7%)	
Unknown	3 (3%)	2 (2.6%)	1 (4.5%)	
**Cancer**				0.279
Lung	15 (15.0%)	14 (17.9%)	1 (4.5%)	
GI tract	24 (24.0%)	16 (20.5%)	8 (36.4%)	
Liver/pancreas	37 (37.0%)	29 (37.2%)	8 (36.4%)	
Breast	3 (3.0%)	1 (1.3%)	2 (9.1%)	
Gynaecology	6 (6.0%)	4 (5.1%)	2 (9.1%)	
Urinary tract	8 (8.0%)	7 (9.0%)	1 (4.5%)	
Lymphoma	1 (1.0%)	1 (1.3%)	0 (0%)	
Head and neck/brain	3 (3.0%)	3 (3.8%)	0 (0%)	
Others	3 (3.0%)	3 (3.8%)	0 (0%)	
**ECOG**				0.666
1	1 (1.0%)	1 (1.3%)	0 (0%)	
2	8 (8.0%)	5 (6.4%)	3 (13.6%)	
3	43 (43.0%)	34 (43.6%)	9 (40.9%)	
4	48 (48.0%)	38 (48.7%)	10 (45.5%)	
**Oral intake**				0.008
Less than a mouthful	47 (47.0%)	31 (39.7%)	16 (72.7%)	
Reduced but more than a mouthful	53 (53.0%)	47 (60.3%)	6 (27.3%)	
**Bowel obstruction**				0.091
Without	75 (75.0%)	62 (79.5%)	13 (59.1%)	
With	25 (25.0%)	16 (20.5%)	9 (40.9%)	
**Hydration amount**	249.02 ± 298.50	116.35 ± 119.03	719.41 ± 266.29	< 0.001
Transfusion				0.647
Not used	93 (93.0%)	73 (93.6%)	20 (90.9%)	
Used	7 (7.0%)	5 (6.4%)	2 (9.1%)	
Antibiotic				0.088
Not used	45 (45.0%)	39 (50%)	6 (27.3%)	
Used	55 (55.0%)	39 (50%)	16 (72.7%)	
Albumin				0.334
Not used	94 (94%)	72 (92.3%)	22 (100%)	
Used	6 (6.0%)	6 (7.7%)	0 (0%)	
**Hospitalisation day**	12.04 ± 6.93	12.55 ± 6.64	10.23 ± 7.78	0.063
**Total GDS**	13.04 ± 2.35	13.10 ± 2.41	12.89 ± 2.23	0.575
**Hospital death**				0.041
No	32 (32.0%)	29 (37.2%)	3 (13.6%)	
Yes	68 (68.0%)	49 (62.8%)	19 (86.4%)	

Data presented as mean ± standard deviation (SD) for continuous variables and frequency (percentage, %) for categorical variables. The *p*-values were calculated using the Wilcoxon rank-sum test for continuous variables and Fisher’s exact test for categorical variables

*NTUH* National Taiwan University Hospital, *Chi-Mei* Chi-Mei Medical Centre, *KMUH* Kaohsiung Medical University Hospital, *GDS* Good Death Scale, *GI* Gastro-intestinal, *ECOG* Eastern Cooperative Oncology Group performance status

The average age of participants was 69.19 ± 12.89 years, with the non-hydration group being significantly older (71.26 ± 11.86 years) than the hydration group (61.86 ± 13.97 years) (*p* = 0.005). The mortality rate in hospital was significantly higher in the hydration group than the non-hydration group (*p* = 0.041). The non-hydration group had a better oral intake condition during admission than the hydration group (*p* = 0.008), and the groups also differed significantly with regards to religion (*p* = 0.015). There were no significant differences in hospital, gender, education level, cancer type, ECOG, marital status, bowel obstruction, blood transfusion, antibiotics use or albumin use between the two groups (*p* > 0.05).

**Fig. 2:**
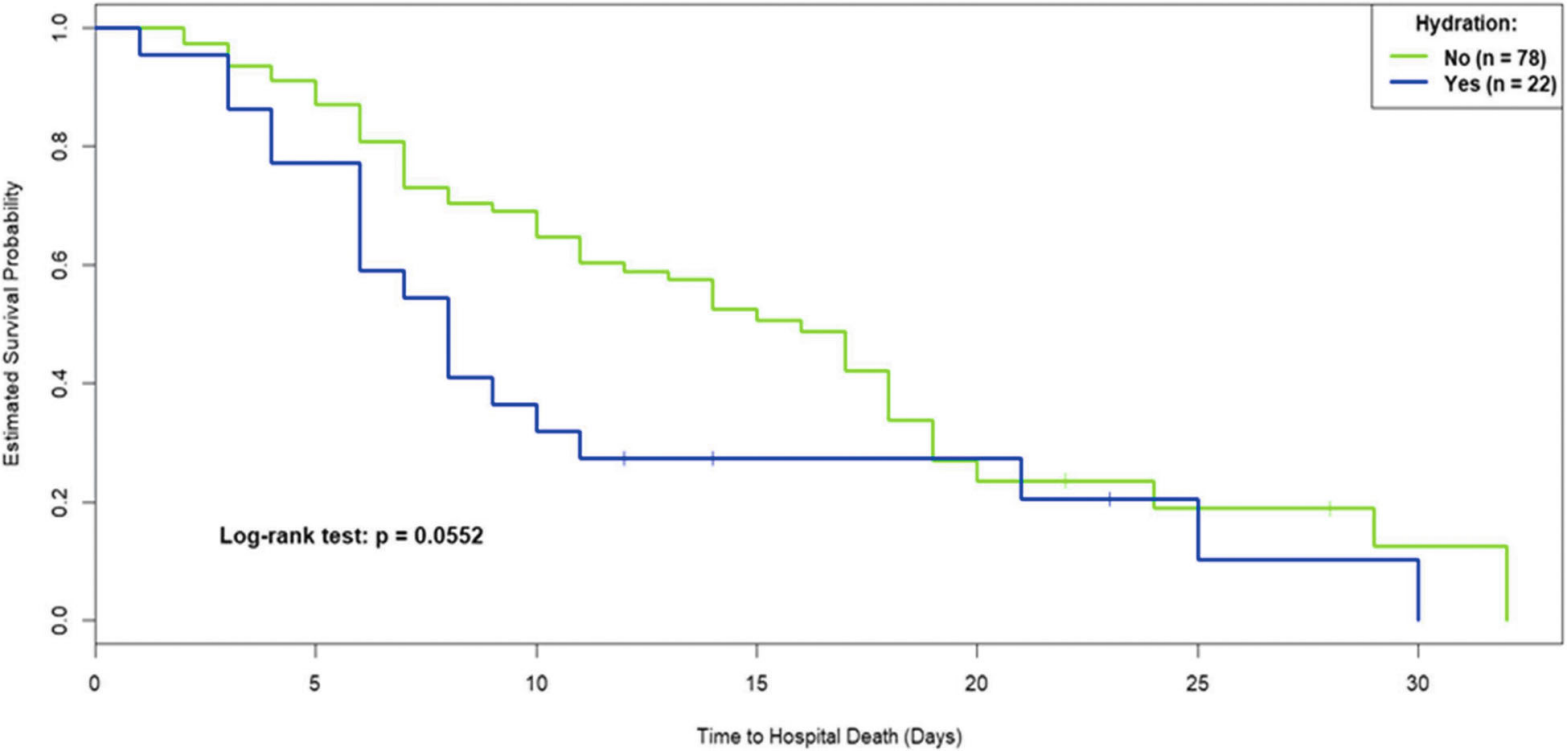
The Survival Curve

The survival analysis (Fig. 2) revealed no significance (*p* = 0.0552) difference in hospital survival time between the non-hydration group and the hydration group. Multivariate analyses of Cox’s proportional hazards analysis of 68 deceased patients was applied to identify the prognostic factors related to mortality and the results are shown in Table 2. The risk of death was higher in those with unknown religion (HR: 9.844, 95% CI: 1.426–67.948) and fatigue or oedema during admission (HR: 1.722, 95% CI: 1.072–2.767, and HR: 1.469, 95% CI: 1.068–2.019, respectively). The hospital, age, education level, oral intake status, artificial hydration amount, other physical symptoms and functional status during admission were not related to the risk of death.

**Table 2:** Multivariate analyses of the predictors of survival status (*n* = 68)

Covariates	Β	Standard Error	z-score	Hazard Ratio	95% Confidence Interval	***p***-value
Institution_NTUH	0.344	0.336	1.054	1.411	0.731-2.724	0.305
Age	− 0.025	0.014	3.211	0.976	0.950-1.002	0.073
ECOG: 3-4 vs 1-2	2.045	1.044	3.838	7.732	0.999-59.839	0.050
Education: 7 ~ 12 vs ≦6	−0.455	0.381	1.424	0.634	0.300-1.340	0.233
Education: > 12 vs ≦6	−0.830	0.482	2.968	0.436	0.170-1.121	0.085
Religion: Buddhism vs Nullifidian	−0.314	0.486	0.417	0.731	0.282-1.893	0.518
Religion: Christian/Catholicism vs Nullifidian	−0.378	0.576	0.431	0.685	0.222-2.118	0.512
Religion: Taoism/Taiwanese folk religion vs Nullifidian	0.208	0.477	0.189	1.231	0.483-3.135	0.664
Religion: Unknown vs Nullifidian	2.287	0.986	5.383	9.844	1.426-67.948	0.020
Oral intake: Less than a mouthful vs Reduced but more than a mouthful	0.245	0.325	0.566	1.277	0.675-2.416	0.452
Hydration: ≧400 vs < 400	0.353	0.368	0.923	1.424	0.692-2.928	0.337
Fatigue admission	0.544	0.242	5.049	1.722	1.072-2.767	0.025
Drowsiness admission	−0.071	0.178	0.161	0.931	0.657-1.319	0.688
Dry admission	−0.083	0.179	0.214	0.921	0.649-1.307	0.643
Myoclonus admission	−0.200	0.373	0.287	0.819	0.394-1.701	0.592
Delirium admission	0.117	0.206	0.322	1.124	0.750-1.684	0.570
Dyspnoea admission	0.316	0.184	2.952	1.371	0.957-1.965	0.086
Oedema admission	0.384	0.162	5.602	1.469	1.068-2.019	0.018

*NTUH* National Taiwan University Hospital, *ECOG* Eastern Cooperative Oncology Group performance status, *GI* Gastro-intestinal

The change in symptoms between day 0 and day 7 in these two groups are shown in Table 3, with no significant change in fatigue, dry mouth, myoclonus, delirium, dyspnoea or oedema. Regarding drowsiness symptoms, both the hydration and the non-hydration groups had more severe symptoms on day 7 than day 0 (*p* = 0.008 and 0.038, respectively), with the hydration group having a greater change in drowsiness than the non-hydration group (*p* = 0.019).

**Table 3:** Changes in symptoms between Day 0 and Day 7 (*n* = 88)

Variable	Non-Hydration (***n*** = 72)			With Hydration (***n*** = 16)			
	Day 0	Day 7	***p***-value	Day 0	Day 7	***p***-value	Between- groups ***p***-value
Fatigue	2.35 ± 1.09	2.52 ± 1.20	0.113	2.60 ± 0.85	3.10 ± 0.96	0.119	0.068
Drowsiness	1.80 ± 1.26	2.12 ± 1.42	0.038	1.81 ± 1.42	2.97 ± 1.12	0.008	0.019
Dry mouth	1.37 ± 1.05	1.27 ± 1.04	0.548	2.00 ± 1.37	1.85 ± 1.38	0.717	0.062
Myoclonus	0.14 ± 0.39	0.21 ± 0.63	0.458	0.19 ± 0.40	0.06 ± 0.25	0.157	0.364
Delirium	0.47 ± 0.86	0.56 ± 0.89	0.182	0.63 ± 0.89	0.63 ± 0.96	0.861	0.690
Dyspnoea	0.63 ± 0.80	0.65 ± 0.83	0.748	0.50 ± 0.73	0.63 ± 0.81	0.414	0.905
Oedema	0.97 ± 0.98	0.97 ± 0.90	0.741	1.13 ± 1.26	1.06 ± 1.00	0.739	0.783

Data are presented as mean ± SD for continuous variables. The *p*-values were calculated using the Wilcoxon rank-sum test for within-group analyses and Mann-Whitney U test for between-groups analyses

Sixty-eight patients died during hospitalisation in the PCUs and logistic regression was applied to analyse the predictors of a good death, as shown in Table 4. A GDS ≧12 indicates a better quality of dying for patients, with only hydration of 86–445 cc significantly associated with a good death (*p* = 0.0011, odds ratio [OR]: 12.8560, 95% CI: 2.774–59.575).

**Table 4:** Multivariate analyses of the predictors of a good death (GDS ≥12, *n* = 68)

Covariates	Β	Standard Error	z-score	Odds Ratio	95% Confidence Interval	***p***-value
***Total score of GDS ≥ 12***						
GI cancer	−1.2134	0.7829	−1.5500	0.2972	0.064-1.379	0.1212
Genitourinary cancer	−2.4362	1.2471	−1.9535	0.0875	0.008-1.008	0.0508
Antibiotic use	1.1199	0.7356	1.5225	3.0644	0.725-12.956	0.1279
Albumin use	−2.0167	1.4147	−1.4255	0.1331	0.008-2.130	0.1540
Religion_ Buddhism	1.8821	1.0184	1.8481	6.5675	0.892-48.340	0.0646
Hydration>86 cc and ≦445 cc	2.5538	0.7824	3.2642	12.8560	2.774-59.575	0.0011

*GI* Gastro-intestinal

## Discussion

This study investigated the effect of AH on the survival period, symptom relief and quality of dying of terminally ill cancer patients, showing that the administration of AH did not prolong survival or improve dehydration symptoms but was associated with a better quality of dying for terminally ill cancer patients.

Morita et al. found that AH did not affect the presence of delirium in terminally ill cancer patients [11]. In a subsequent study, however, the administration of intravenous AH worsened fluid retention symptoms in terminal lung and gastric cancer patients. Reducing the volume of intravenous hydration improved fluid retention symptoms without any deterioration of dehydration symptoms [12]. In terminal patients with abdominal malignancies, patients given 1 L or more AH per day, although they had lower dehydration scores than those who received less than 1 L AH, had higher symptom scores for oedema, ascites and pleural effusion [13]. Nakajima et al. also reported that the symptom scores for oedema, ascites and bronchial secretion were higher in patients who received more than 1 L of AH per day [22], whereas Bruera et al. found no difference in dehydration symptoms, such as fatigue, myoclonus, drowsiness and delirium, 4 days later between patients who received 1 L or 100 ml normal saline per day [10]. Our study showed no significant change in fatigue, delirium, dry mouth or myoclonus after 1 week between the hydration and non-hydration groups. Furthermore, the drowsiness level was more severe in the hydration group. In our study, we used 400 mL as the cut-off point to separate hydration or not, whereas previous studies used 1 L as the cut-off point and the groups who received over 1 L AH per day had lower dehydration scores but more fluid retention symptoms. Therefore, giving less than 1 L or even less than 400 ml AH per day does not affect the dry mouth or myoclonus symptoms or exacerbate the severity of oedema or dyspnoea in terminally ill cancer patients after 1 week. In previous studies, many symptoms of terminally ill cancer patients had little relationship to AH [10, 23–25], thus routine AH is not recommended for the treatment of terminally ill cancer patients’ symptoms.

This study also found that the administration of AH to terminally ill cancer patients did not influence survival, similar to previous studies [6, 10]. According to Torres-Vigil, African Americans, Latinos and Asian Americans are more likely than non-Hispanic white subjects to view AH as food or as both food and medicine. Indeed, in a previous study, most terminally ill cancer patients’ families regarded AH as basic care and wanted continuous AH administration in the hope that the patient’s condition would improve [5–7, 16, 17]. Chiu et al. found that most terminally ill cancer patients in the PCU wish to use ANH and want AH, as they and their families believed AH could help patients avoid dehydration or starvation or prevent them from starving to death. Also, some patients believe ANH could prolong all patients’ life [26]. Huang et al. also found that withdrawing ANH was a difficult decision for families during end-of-life care [27]. In Taiwan, a culture where food intake is strongly related to healing and hope, AH is regarded as a “lifeline”, thus withholding or withdrawal of AH is often mistakenly regarded as unethical by those who do not understand the role of AH in terminally ill cancer patients in the stage of actively dying. Many physicians prescribe AH to allay the fears of family members that the patient might be “starved to death.” Once again, our study demonstrated that AH does not prolong a patient’s life, so instead of focusing on the patient’s intake, healthcare professionals should explain to families the role of AH during the terminal stages.

Nevertheless, appropriately administering AH to terminally ill cancer patients could achieve a better quality of dying. In the United States, Cohen et al. found that terminally ill patients and their families believed hydration could bring hope, improve patients’ symptoms and enhance QOL [28]. Previous studies which only measure the influence of AH on QOL found no such remarkable effect [10], however, QOL is not equivalent to the quality of dying, which may be influenced by many other factors than those found in QOL. In our study, appropriate hydration was a predictor of better GDS (GDS≧12). Furthermore, as in many other studies, appropriate hydration may meet the psychological needs and expectations of terminally ill cancer patients and their families [5–7, 16, 17] by reducing the burden of making difficult decisions and helping both patients and their families to better prepare to face death. Nevertheless, more research is warranted to validate the impact of AH on the quality of dying of terminally ill cancer patients.

This study was a pilot prospective, multi-centre, observational project and the recruited subjects were from different hospitals in northern and southern Taiwan. While the study may be representative of the national cancer patient population, there were several study limitations. First, the number of study subjects was small, so future studies should involve more patients to confirm the effect of hydration on terminally ill cancer patients. Second, the imbalance between groups showed that fewer terminally ill cancer patients in Taiwan receive AH, hence there is a risk of sample bias related to the selection of patients referred for palliative care. Third, this study was not blinded, hence, the clinical assessors may have had some preconceived bias. A randomised controlled trial to decrease the bias of statistical analysis and the placebo effect in the future is warranted. Fourth, we did not record the indication of hydration, whether it was mainly under patient/family desire, or physician-led, this should be considered in future studies. Fifth, it was not possible to collect detailed data of median survival from hydration to death in each group as some patients survived and were discharged from PCUs, hence, were not followed up. However, this study only evaluated the hydration effect of survival status in the hospital, not the whole survival condition. In future, patients could be followed up until death, even if they are discharged. Finally, the two groups of patients were not comparable in terms of the characteristics of age, education and religion. Nevertheless, we performed regression analysis to adjust for these differences. This is a pilot study conducted in Asia, and a large-scale, cross-cultural, multi-centre study is ongoing based on the results of this pilot study.

## Conclusions

For terminally ill cancer patients in PCU, AH over 400 mL might not prolong survival nor significantly improve the dehydration symptoms, but appropriate AH may improve the quality of dying. Hydration remains an ethical dilemma, especially in the Asian context. Communication with patients and their families is recommended regarding the benefit and adverse effects of AH, as this may help better prepare them for the final stage of life and achieve a good death. In the future, a large-scale randomised-controlled study of the impact of AH on the quality of dying is warranted.

## Data Availability

The datasets used and analysed in the current study are available from the corresponding author on reasonable request.

## Abbreviations

QOL: Quality of life
AH: Artificial hydration
ANH: Artificial nutrition and hydration
PCU: Palliative care units
GDS: Good death scale
ECOG: Eastern Cooperative Oncology Group.
IPOS: Integrated Palliative care Outcome Scale
MDAS: Memorial Delirium Assessment Scale
HR: Hazard ratio
CI: Confidence Interval
OR: Odds ratio
